# Sepsis-Associated Acute Kidney Injury: Pathophysiology and Treatment Modalities

**DOI:** 10.7759/cureus.75992

**Published:** 2024-12-19

**Authors:** Martin Gerardo Aguilar, Hassen A AlHussen, Prenika Devadas Gandhi, Priyadeep Kaur, Mounica A Pothacamuri, Mariam Altaf Husain Talikoti, Nandita Avula, Pallavi Shekhawat, Alisson Barbosa Silva, Arshpreet Kaur, Manju Rai

**Affiliations:** 1 Internal Medicine, Garci︠a PCP Universidad de Durango Campus Ciudad Jua︠rez, Chihuahua, MEX; 2 Critical Care Medicine, Sulieman Alhabib Medical Academy, Riyadh, SAU; 3 Internal Medicine, Metropolitan University College of Medicine, Antigua, ATG; 4 Internal Medicine, Punjab Institute of Medical Sciences, Jalandhar, IND; 5 Internal Medicine, Trinity West Medical Center, Steubenville, USA; 6 Internal Medicine, Kanachur Institute of Medical Sciences, Mangaluru, IND; 7 Internal Medicine, Sri Ramachandra Institute of Higher Education and Research, Chennai, IND; 8 Obstetrics and Gynecology, Employees State Insurance-Post Graduate Institute of Medical Sciences and Research Delhi, Delhi, IND; 9 Internal Medicine, UNIME School of Medicine, Bahia, BRA; 10 Surgery, School of Medical Sciences and Research, Sharda University, Greater Noida, IND; 11 Biotechnology, Shri Venkateshwara University, Gajraula, IND

**Keywords:** cytokine storm, emerging therapies, endothelial activation, fluid management, inflammatory response, microcirculatory dysfunction, oxidative stress, renal replacement therapy, s-aki pathophysiology, sepsis-associated acute kidney injury

## Abstract

Sepsis-associated acute kidney injury (S-AKI) is a critical complication that significantly contributes to the morbidity and mortality of sepsis patients. This narrative review explores the complex and multifactorial pathophysiology of S-AKI, which involves hemodynamic alterations, microcirculatory dysfunction, endothelial damage, inflammatory responses, oxidative stress, and direct tubular injury. Conventional perspectives linking S-AKI primarily to reduced renal blood flow are now being reconsidered, with growing insights highlighting the significance of microcirculatory dysfunction and endothelial activation as key contributors. The review also discusses the current diagnostic approaches for S-AKI, emphasizing the limitations of existing biomarkers and the need for earlier and more accurate detection methods. Standard treatment strategies focus on supportive care, including fluid management, vasopressor therapy, and renal replacement therapy. However, these approaches often fail to address the underlying mechanisms of S-AKI, resulting in persistently high mortality rates. Emerging therapies, including the use of antioxidants, anti-inflammatory agents, and stem cell-based treatments, offer the potential for improved outcomes. These innovative approaches aim to target the pathophysiological processes at the molecular level, offering hope for better management of S-AKI. The review highlights the need for ongoing research to further understand the mechanisms driving S-AKI and to develop more effective therapeutic strategies.

## Introduction and background

Sepsis, a life-threatening condition resulting from a dysregulated immune response to infection, is a major cause of morbidity and mortality worldwide. Acute kidney injury (AKI) is a frequent and severe complication of sepsis, occurring in up to 50% of critically ill patients with septic shock [1]. Sepsis-associated acute kidney injury (S-AKI) is characterized by a sudden decline in kidney function, leading to the accumulation of waste products, electrolyte imbalances, and fluid overload [1-2]. The pathogenesis of S-AKI is complex and multifactorial, involving hemodynamic alterations, endothelial and microcirculatory dysfunction, inflammatory responses, oxidative stress, and direct tubular cell injury [1-3].

Traditional views attributed S-AKI primarily to decreased renal blood flow; however, recent studies have revealed that global renal blood flow may remain unchanged or even increase in sepsis, challenging conventional wisdom. Instead, microcirculatory dysfunction, endothelial activation, and a dysregulated immune response play critical roles in the development of S-AKI [4-5]. The inflammatory cascade triggered by sepsis leads to a cytokine storm, neutrophil activation, and mitochondrial dysfunction, all of which contribute to renal damage [6-7].

Despite advancements in understanding the pathophysiology of S-AKI, its management remains challenging. Current treatment strategies focus on supportive care, including fluid management, vasopressor therapy, and renal replacement therapy. However, these interventions often do not address the underlying pathophysiological mechanisms, and mortality rates remain high. Emerging therapies, such as the use of antioxidants, anti-inflammatory agents, and stem cell-based treatments, are being explored to improve outcomes in S-AKI patients.

The objective of this narrative review is to provide a comprehensive overview of the pathophysiology of sepsis-associated acute kidney injury, highlight current diagnostic and treatment strategies, and explore emerging therapies that hold promise for better management and outcomes in S-AKI.

## Review

Pathophysiology of S-AKI

S-AKI involves several complex mechanisms, including hemodynamic alterations, endothelial dysfunction, microcirculatory dysfunction, inflammatory responses, oxidative stress, and tubular cell injury.

Hemodynamic Alterations

Sepsis induces profound alterations in systemic hemodynamics, characterized by initial hyperdynamic circulation followed by hypodynamic circulation [1]. In the early stages, increased cardiac output and decreased systemic vascular resistance lead to hypotension [2]. Contrary to traditional beliefs, recent studies suggest that global renal blood flow may be preserved or even increased during sepsis, despite the development of AKI [3]. This paradox highlights the complexity of sepsis-induced hemodynamic changes and suggests that factors beyond macro-hemodynamics play crucial roles in AKI development.

Microcirculatory Dysfunction

Microcirculatory dysfunction is a hallmark of S-AKI [4]. This dysfunction arises from several mechanisms. Sepsis triggers endothelial activation, leading to increased vascular permeability, leukocyte adhesion, and microvascular thrombosis [5]. Additionally, altered vasoregulation disrupts the balance between vasodilators, such as nitric oxide, and vasoconstrictors, like endothelin-1, resulting in heterogeneous microvascular blood flow [6]. The degradation of the endothelial glycocalyx, a structure critical for vascular integrity, further compromises microcirculatory function during sepsis [7]. Furthermore, capillary shunting can occur, causing preferential blood flow through larger vessels and bypassing smaller capillaries, which leads to tissue hypoxia despite seemingly adequate global perfusion [8]. These microcirculatory alterations result in regional hypoperfusion, tissue hypoxia, and cellular dysfunction, which collectively contribute to the development of AKI [9].

Inflammatory Response and Immune Activation

**Figure 1:**
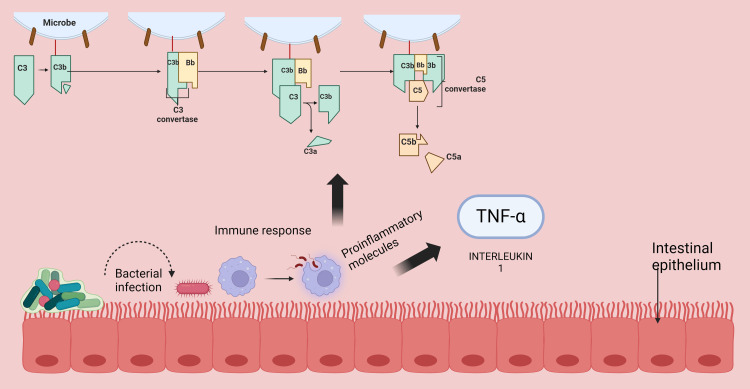
A bacterial infection in intestinal tissue triggers an immune response mediated by macrophages, monocytes, and complement activation. This leads to vasodilation and the secretion of proinflammatory molecules such as TNF-α and IL-1. TNF: tumor necrosis factor; IL: interleukin (Image credits: Arshpreet Kaur)

The immune system serves as the body's primary defence against pathogens during sepsis [1] (Figure 1). This process activates cells like macrophages and lymphocytes, which release various inflammatory mediators, including cytokines (e.g., tumor necrosis factor (TNF)-α, interleukin (IL)-1, IL-2, IL-6, IL-8) and chemokines [10-12].

**Figure 2:**
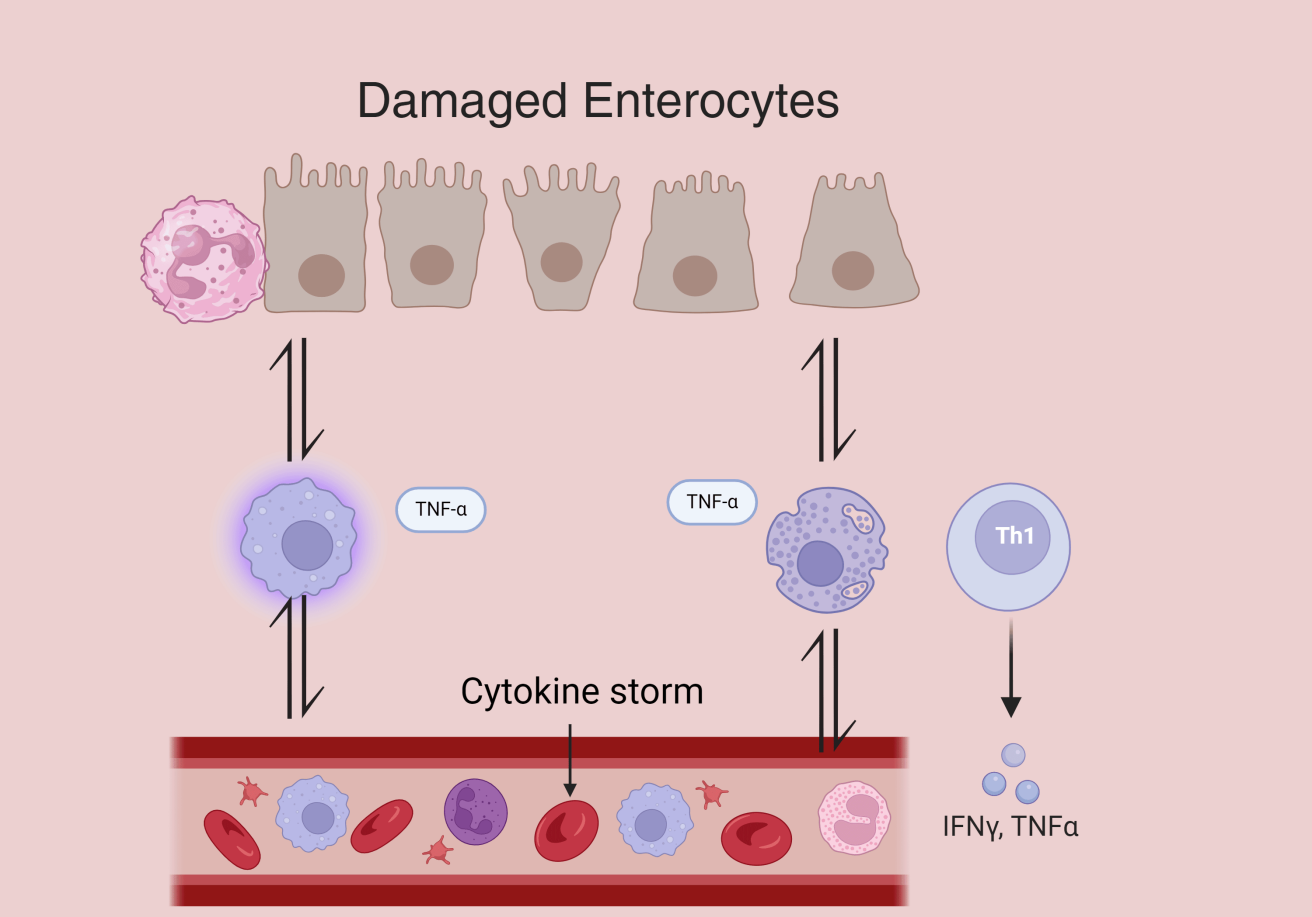
Proinflammatory cytokines induce vasodilation and extravasation of polymorphonuclear leukocytes and other cytokines, which target the infection. However, in the presence of a dysregulated immune response, these mechanisms can lead to a cytokine storm and a subsequent procoagulant state. TNF: tumor necrosis factor; IFN-γ: interferon gamma; Th1: T-helper 1 cells (Image credits: Alisson Barbosa Silva)

Cytokine Storm: In severe sepsis, a cytokine storm can occur, characterized by an amplified release of pro-inflammatory cytokines (Figure 2). Immune cells detect pathogen-associated molecular patterns (PAMPs) and damage-associated molecular patterns (DAMPs) via pattern recognition receptors (PRRs), such as Toll-like receptors (TLRs), present on renal tubular epithelial cells (TECs) [10-12].

Neutrophil Activation and Infiltration: Neutrophils, essential to the innate immune system, are activated and infiltrate renal tissue in S-AKI [13]. They release proteases, reactive oxygen species (ROS), myeloperoxidase (MPO), and other cytotoxic agents, which can directly damage endothelial and renal tubular cells [10,11,14].

Oxidative Stress and Mitochondrial Dysfunction

Sepsis induces oxidative stress and mitochondrial dysfunction in renal cells. Receptor-interacting protein kinase-3 (RIPK3) exacerbates AKI in sepsis by promoting oxidative stress and mitochondrial dysfunction through nicotinamide adenine dinucleotide phosphate hydrogen (NADPH)** **oxidase-4 (NOX4) upregulation and inhibition of mitochondrial complexes I and III [15].

5. Tubular Cell Injury

**Figure 3:**
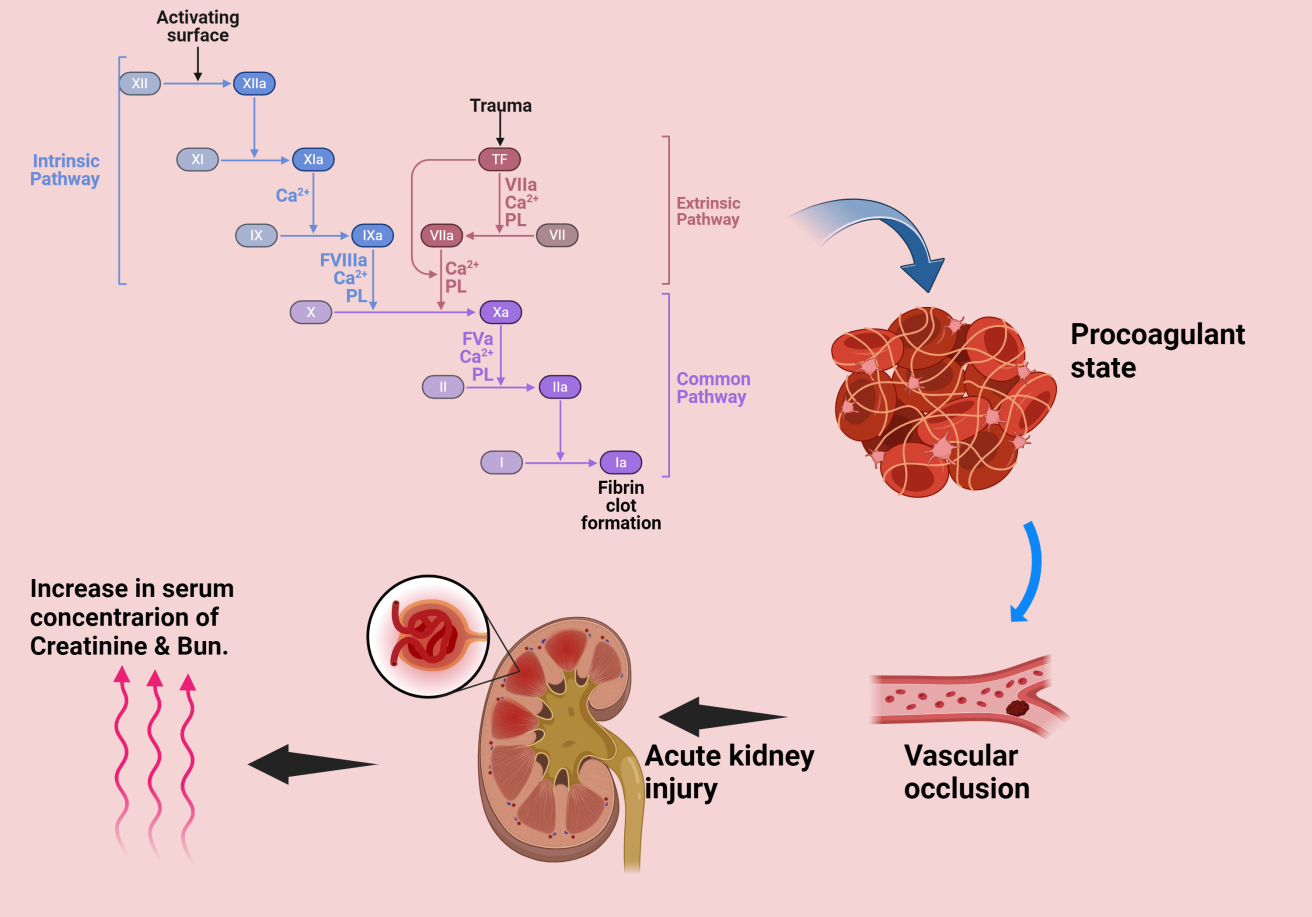
The procoagulant state associated with sepsis can damage or obstruct blood vessels, compromising systemic and renal circulation. This reduction in glomerular filtration rate (GFR) results in elevated serum creatinine and blood urea nitrogen (BUN), presenting clinically as Acute Kidney Injury (AKI) linked to a dysregulated immune response. TF: Tissue Factor; VIIa: Activated Factor VII; Ca²⁺: Calcium ions; PL: Phospholipids; FVIIIa: Activated Factor VIII; FVa: Activated Factor V; Xa: Activated Factor X; II: Prothrombin; IIa: Thrombin (activated Prothrombin); I: Fibrinogen; Ia: Fibrin (activated Fibrinogen); BUN: Blood Urea Nitrogen.   (Image credits: Pallavi Shekhawat)

Platelet-derived transthyretin (TTR) has been shown to play a role in kidney damage during sepsis (Figure 3). Studies using ticagrelor [16] have revealed TTR's effects on human renal proximal tubule epithelium (HK2) cells, increasing mRNA and protein levels of protein kinase B, phosphatidylinositol 3-kinase, and extracellular regulated protein kinase (ERK).

Diagnosis and staging of S-AKI

Recent research has focused on developing novel diagnostic approaches for AKI in septic patients. AKI is a common complication, affecting up to 70% of sepsis cases [17]. The Kidney Disease Improving Global Outcomes (KDIGO) guidelines, last updated in 2012, categorize S-AKI into early (≤48 hours) and late (≤7 days) stages (Table 1) [17-18].

**Table 1:** KDIGO Classification 2012 KDIGO, systems for AKI classification; KDIGO, Kidney Disease Improving Global Outcomes

Stage	Serum Creatinine	Urine Output
1	1.5–1.9 times baseline OR X0.3mg/dl (X26.5mmol/l) increase	0.5ml/kg/h for 6–12 hours
2	2.0–2.9 times baseline	0.5ml/kg/h for X12 hours
3	3.0 times baseline OR Increase in serum creatinine to X4.0mg/dl (X353.6mmol/l) OR Initiation of renal replacement therapy OR, In patients o18 years, decrease in eGFR to o35ml/min per 1.73 m2	0.3ml/kg/h for X24 hours OR Anuria for X12 hours

While KDIGO criteria, which include serum creatinine (SCr), estimated glomerular filtration rate (eGFR), and urine output, are widely used in clinical practice, their effectiveness as sole diagnostic markers has been questioned due to limitations in early detection [19-20]. This highlights the critical need for improved early diagnostic methods, as S-AKI often progresses silently. Timely diagnosis is crucial for optimizing patient outcomes and survival rates [21].

In the diagnosis of S-AKI, various biomarkers are gaining acceptance, particularly cell cycle arrest markers [22]. Several biomarkers have shown strong correlations with S-AKI, including urine and serum neutrophil gelatinase-associated lipocalin (NGAL), urinary interleukin-18 (IL-18), kidney injury molecule-1 (KIM-1), Netrin-1, sCD163, serum estradiol levels, and serum soluble thrombomodulin [23].

A meta-analysis by Pan et al. compared the diagnostic efficacy of multiple biomarkers for S-AKI, including NGAL, KIM-1, liver-type fatty acid-binding protein (L-FABP), IL-18, and tissue inhibitor of metalloproteinases-2 (TIMP-2) × insulin-like growth factor-binding protein-7 (IGFBP-7). Among these, serum or urinary NGAL demonstrated the highest diagnostic accuracy in predicting S-AKI (Table 2) [24]. 

**Table 2:** Descriptive comparison between biomarkers in performance with different populations for detecting S-AKI. S-AKI: Sepsis Associated Acute Kidney Injury; NGAL: neutrophil gelatinase-associated lipocalin; IL-18: interleukin-18; Cr: urine creatinine; KIM-1: kidney injury molecule-1; L-FABP: liver-type fatty acid-binding protein; TIMP-2 × IGFBP-7: tissue inhibitor of metalloproteinases-2 × insulin-like growth factor-binding protein-7

Population	Best performing biomarkers	Details
Whole population	sNGAL, uNGAL	Both have the best diagnostic accuracy with early onsent within (48hr)
Critical Patients	NGAL/Cr, L-FABP, TIMP-2XIGFBP-7	No distinguishment with good predictive values
Non-critical patients/Medical	sNGAL, uNGAL,KIM-1	Superior to IL-18
Surgical patients	NGAL/Cr, KIM-1	Best diagnostic accuracy

Furthermore, elevated levels of urinary TIMP-2 × IGFBP7 have been associated with lower 30-day survival rates [25]. Conversely, when the KDIGO bundle was supplemented with urinary TIMP-2 × IGFBP7 assessment, a reduced incidence of moderate-to-severe AKI was observed [26].

Treatment modalities

Supportive Care and Prevention

Effective fluid management in sepsis is crucial for preventing and managing AKI. It should be tailored to address individual clinical factors and comorbidities and adhere to evidence-based guidelines. Research has demonstrated that the use of balanced crystalloids versus normal saline for fluid resuscitation in septic patients is associated with reduced mortality, lower risk of AKI, decreased necessity for renal replacement therapy (RRT), and shorter stays in the intensive care unit (ICU) [27].

Fluid volume is a critical aspect to consider when formulating resuscitation strategies involving vasopressor use and developing individualized treatment plans for managing sepsis-related AKI in various patient populations [28]. A retrospective chart review indicated that a positive fluid balance associated with fluid resuscitation in sepsis correlates with worse outcomes, highlighting the necessity of careful fluid management to optimize patient outcomes and prevent complications [29].

AKI in sepsis is associated with prolonged hospital stays, increased mortality, and overall higher healthcare costs. A review of S-AKI discusses the intricate mechanisms such as hemodynamic instability resulting from inflammation, oxidative stress, and shock that contribute to decreased renal perfusion and ischemia. Effective management of hypotension in these settings targets the improvement of renal microcirculation and overall function [30]. Patients with a history of hypertension are found to be particularly vulnerable to drastic blood pressure fluctuations, which significantly affect renal outcomes [30]. Vigilant blood pressure management, along with the judicious use of fluids, vasopressors, and renal replacement therapy, is critical for renal recovery and overall prognosis [31-32].

The impact of fluid overload on mortality in S-AKI patients undergoing continuous renal replacement therapy (CRRT) was examined in a study. This research identified optimal cutoff values for predicting 28-day mortality based on fluid overload: greater than 4.6% before CRRT initiation and greater than 9.6% total fluid overload. Patients exceeding these thresholds had significantly higher mortality rates [32]. This finding highlights fluid overload as a predictor of mortality and suggests that managing fluid overload through CRRT may improve survival outcomes.

A review of high-volume haemofiltration (HVHF) in sepsis treatment concluded that HVHF did not significantly reduce mortality compared to standard renal replacement therapies [33]. Although HVHF improved hemodynamic parameters and reduced inflammatory markers, these benefits did not necessarily translate to a clear survival advantage, emphasizing the need for further research to assess HVHF's effectiveness in sepsis [33].

An observational cohort study explored the role of peripheral perfusion indicators, such as capillary refill time (CRT) and peripheral perfusion index (PI), in managing S-AKI [34]. The study found that both CRT and PI abnormalities were associated with higher 28-day mortality rates, with PI being a strong early predictor of death. A weak correlation was also noted between PI and fluid balance, suggesting that while peripheral perfusion does not intrinsically differ between S-AKI and non-S-AKI patients, it serves as a prognostic marker for mortality influenced by fluid balance.

The altered pharmacokinetics in AKI necessitate careful selection of broad-spectrum antibiotics. Guidelines recommend antibiotics such as piperacillin-tazobactam or cefepime for broad Gram-negative coverage, and vancomycin or linezolid for Gram-positive (particularly methicillin-resistant *Staphylococcus aureus *(MRSA)) coverage, with appropriate renal dose adjustments [35]. For severe sepsis or suspected polymicrobial infections, combination therapy using agents with different spectra is considered, while closely monitoring kidney function to prevent toxicity [36].

A retrospective cohort study comparing vancomycin/piperacillin-tazobactam versus vancomycin/cefepime in sepsis patients with pre-existing AKI found no significant differences in maximum serum creatinine levels, AKI progression, dialysis requirements, or major adverse kidney events between the two regimens (p=0.459 for serum creatinine; p=0.895 for AKI progression) [30]. This suggests that vancomycin/piperacillin-tazobactam does not worsen renal outcomes compared to vancomycin/cefepime. Additionally, it has been found that daptomycin and linezolid are alternatives to vancomycin that may reduce the risk of S-AKI [36].

A prospective, multicenter observational study examined the timing of antipseudomonal β-lactam dose adjustments in sepsis patients with AKI [37]. Patients receiving late dose adjustments (late β-lactam antibiotic (L-BLA)) after 24 hours of sepsis recognition showed significantly lower in-hospital mortality compared to those with early adjustments (early β-lactam antibiotic (E-BLA), with a hazard ratio of 0.588 (95% CI, 0.355-0.974), indicating a mortality benefit associated with later dose adjustments.

The ongoing Antibiotic Choice On Renal Outcomes (ACORN) trial, a randomized, non-blinded study, is investigating the effects of antipseudomonal cephalosporins (e.g., cefepime) versus penicillins (piperacillin-tazobactam) on renal outcomes in acutely ill patients with gram-negative infections [38]. This study aims to assess AKI severity and mortality within 14 days using an unadjusted proportional odds regression model.

Research on serum vancomycin concentrations revealed that elevated levels are associated with an increased risk of nephrotoxic AKI in critically ill patients and were predictive of nephrotoxic injury [39]. This emphasizes the importance of careful monitoring and dose adjustment of vancomycin to mitigate AKI risk. In contrast, a study on azithromycin showed minimal effects on major adverse kidney events (MAKE) in critically ill patients with sepsis-associated AKI, suggesting its safe use in this population [40].

Pharmacological Interventions

Pharmacological interventions in S-AKI have been the focus of several studies, exploring the outcomes of treatment with anti-oxidant, anti-inflammatory, and renal vasodilators. S-AKI is characterized by decreased creatinine clearance and renal antioxidant activity, coupled with enhanced oxidative stress and elevated renal mRNA levels of TNF-α, IL-1β, and transforming growth factor beta (TGF-β). Brazilian green propolis (GP) has shown promise in S-AKI treatment due to its anti-inflammatory, antioxidant, and immunomodulatory properties [41]. In experiments with male Wistar rats, GP treatment combined with volume expansion and antibiotic therapy resulted in a 70% improvement in survival rates. GP protects renal mitochondrial morphology by decreasing terminal deoxynucleotidyl transferase-mediated deoxyuridine triphosphate. It also attenuates the upregulation of Toll-like receptor 4/nuclear factor-kappa B (TLR-4/NF-kappa B), inflammatory cytokine levels, and macrophage infiltration typically observed in sepsis. Additionally, GP increases reduced glutathione and restores renal tubular function, as evidenced by increased mean glomerular filtration rate (GFR).

Another promising avenue of research involves the use of 6-gingerol (6G) and 10-gingerol (10G) in S-AKI treatment [42]. These compounds have demonstrated an increase in GFR and decreases in serum creatinine (SCr) and blood urea nitrogen (BUN) levels, as well as urinary protein overload. The mechanisms underlying these effects include free radical scavenging, repression of nitric oxide (NO) metabolism, and reduction of cytokine expression upregulation in kidney tissue. Antioxidant effects were evident through decreased malondialdehyde and increased glutathione (GSH) activity. Nuclear magnetic resonance analysis has also detected increases in dimethylamine and methylsulfonylmethane metabolites in septic animals treated with 6G or 10G.

Nitric oxide (NO) plays a crucial role in the mechanisms causing AKI in sepsis [43-46]. The kidney expresses three nitric oxide synthase (NOS) isoforms: endothelial (eNOS), found in kidney vessels and glomeruli, neuronal (nNOS), found in the efferent arteriole and macula densa, and inducible (iNOS). While eNOS and nNOS are the main sources of NO under basal conditions and participate in renal hemodynamic regulation, iNOS is dramatically increased in sepsis. Overexpression of iNOS in the renal cortex causes blood shunting to this region, leading to medullary ischemia. Large amounts of iNOS-derived NO may also inhibit constitutively expressed NOS activity, affecting renal microcirculation and impairing microvascular homeostasis and renal function. However, small amounts of NO are essential for preserving GFR in endotoxemic shock due to its vasodilatory effect on the afferent arteriole and antithrombotic properties.

Studies evaluating therapeutic strategies that increase eNOS expression/activity through indirect inhibition of iNOS have shown beneficial effects in S-AKI. For instance, treatment with erythropoietin (EPO) and blood transfusions improved renal function in rats with S-AKI by restoring eNOS. Resveratrol (RES), a polyphenolic nutraceutical with vasodilatory and antioxidant activities, has also shown promise in S-AKI treatment [47]. When administered at 6, 12, and 18 hours post cecal ligation and puncture (CLP) in a murine model of S-AKI, resveratrol significantly improved capillary perfusion, increased renal blood flow, and enhanced glomerular filtration rate without raising systemic pressure. This dual mechanism of action, restoring renal microcirculation and scavenging reactive nitrogen species, protects the tubular epithelium even when administered after the onset of sepsis.

Recent clinical trials have demonstrated the efficacy of bovine-derived alkaline phosphatase (biAP) in S-AKI treatment [48]. biAP administration resulted in decreased plasma creatinine levels, reduced urinary excretion of glutathione-S-transferase A1, and attenuation of iNOS upregulation. While the exact mechanism remains unclear, it is likely related to the dephosphorylation and detoxification of detrimental molecules involved in S-AKI pathogenesis. For example, endotoxin (lipopolysaccharide) becomes non-toxic after dephosphorylation, and adenosine triphosphate, a proinflammatory mediator, can be converted into an anti-inflammatory molecule by biAP. Table 3 summarizes the various pharmacological interventions and their outcomes and gaps.

**Table 3:** Summary of pharmacological interventions in sepsis-associated acute kidney injury (S-AKI)

Study	OBJECTIVES	INTERVENTIONS	OUTCOMES	GAPS
Silveira et al.,2021 [41]	Anti-inflammatory, Antioxidant, and Immunomodulatory properties of Brazilian green propolis (GP) in S-AKI	Research done in rats by inducing sepsis by cecal ligation and puncture (CLP). Male Wistar rats were divided into groups—control (sham-operated); CLP (CLP only); and CLP + GP (CLP and treatment with GP at 6 h	The survival rate was 70% in the CLP + GP group and 40% in the CLP group GP reduced oxidative stress, modulate inflammation, and preserve endothelial function of kidney.	None
Rodrigues et al., 2018 [42]	Treatment with 6-gingerol (6G) and 10-gingerol (10G) in S-AKI	Research done in rats by inducing sepsis by cecal ligation and puncture (CLP). Male rats were divided into groups—control (sham-operated); CLP (CLP only); and CLP + 6G or 10G (CLP and treatment with 6G or 10G (25 mg/kg) by injecting intraperitoneally.	Gingerols attenuated septic acute kidney injury (AKI) by decreasing renal disturbances, oxidative stress, and inflammatory response through a mechanism possibly correlated with increased production of dimethylamine and methylsulfonylmethane. Thus improving the survival rate.	The ability of gingerols to affect some important catabolic pathways remains to be elucidated.
Santos et al., 2013 [46]	Role of Nitric oxide in S-AKI	A study performed in rats infused with lipopolysaccaride (LPS)	The protective effects of the indirect inhibition of inducible nitric oxide synthase (iNOS) and increased endothelial nitric oxide synthase (eNOS) expression activity indicated beneficial effects in S-AKI.	Is there a time window in S-AKI in which selective iNOS inhibition would be protective?
Holthaff et al., 2012 [47]	Use of Resveratrol (a polyphenol vasodilator) in S-AKI	Studies done by using the cecal ligation and puncture (CLP) murine model of S-AKI.	Improved survival rate by dual mechanism of action: restore the renal microcirculation (increase renal blood flow (RBF) and glomerular filtration rate (GFR)) and scavenge reactive nitrogen species.	Unfortunately, effective therapy in the septic patient is hampered because in this study, we examined the acute effects of resveratrol (RES) on CLP-induced AKI (i.e therapy usually begun only after the onset of symptoms)
Peters et al., 2014 [48]	The potential of Alkaline phosphate as treatment in S-AKI.	Studies were carried out till phase IIa clinical trial with relatively small number of patients.	Has showed renal protective effect through detoxification of LPS and/or adenosine triphosphate (ATP)	The rationale behind the renal protective effects remains to be fully elucidated

Renal Replacement Therapy (RRT)

The Kidney Disease: Improving Global Outcomes (KDIGO) guidelines outline several indications for RRT in patients with AKI. These include severe acid-base disorders such as persistent metabolic acidosis, electrolyte imbalances like refractory hyperkalemia, the presence of uremic symptoms, oliguria or anuria, and fluid imbalances such as edema or overload refractory to diuretic support [49]. In cases of compromised hemodynamics, continuous renal replacement therapy (CRRT) is the treatment of choice for AKI in intensive care unit settings, regardless of the presence of sepsis.

The history of CRRT dates back to 1977 in Germany when Peter Kramer accidentally inserted a catheter into the femoral artery while attempting to initiate hemodialysis. He discovered that blood flow driven by mean arterial pressure provided both ultrafiltration and hemofiltration due to the difference in arteriovenous pressures. This technique was termed continuous arteriovenous hemofiltration (CAVH) [50]. CRRT has since evolved into an extracorporeal blood purification system and is now considered the primary form of renal replacement therapy in ICU settings. It is prescribed and implemented over 24 hours to several days for critically ill adult and pediatric patients [50].

CRRT operates on four physiological principles: diffusion, ultrafiltration, convection, and adsorption. It can be administered through four different modalities: slow continuous ultrafiltration, continuous veno-venous hemofiltration, continuous veno-venous hemodiafiltration, and continuous veno-venous hemodialysis [50]. CRRT is known for its efficiency in gently removing fluid compared to intermittent hemodialysis or slow low-efficiency dialysis. It provides continuous kidney support by slowly removing fluid, urea, and other solutes, which reduces the risk of cerebral edema. This makes it particularly appropriate for patients who develop intracranial pressure in the ICU [50].

The timing of CRRT initiation has been a subject of debate. Wang et al. reported that pooled results from cohort studies and controlled trials indicated a higher 28-day mortality rate in patients who received delayed renal replacement therapy compared to those who received early therapy [51]. While there is no universally accepted definition of early or late initiation, several studies reviewed by Fonseca et al. have described the benefits of early CRRT initiation [52]. These benefits include the avoidance of hypervolemia and more adequate acid-base balance, potentially leading to lower mortality rates and enhanced kidney recovery. Early initiation is generally understood to occur when patients present with low scores on the Risk, Injury, Failure, Loss, and End-stage kidney disease (RIFLE), KDIGO, or Acute Kidney Injury Network (AKIN) classifications.

However, the debate on timing continues, as Agapito et al. also noted that more recent high-evidence studies, such as meta-analyses and randomized controlled trials, show no significant difference in survival rates between early and late initiation of renal replacement therapy [52].

Emerging Therapies

Extracorporeal therapies aim to remove cytokines from the blood and serve as a therapeutic tool against sepsis [53]. These techniques enable correction by directly affecting the molecular and electrolyte composition of blood. However, a lack of consensus on guidelines leads to heterogeneous practices globally [54]. A survey conducted in 200 hospitals across China found that CRRT is the most common extracorporeal therapy used for AKI (99%) in critically ill patients, followed by plasma exchange (37%) and intermittent hemodialysis (25%) [53]. Zarbock et al., in their consensus statement, suggested that extracorporeal blood purification in sepsis can be considered for immunomodulatory support in patients meeting specific time and/or biological criteria [54].

Cell-based therapies, such as mesenchymal stem cells (MSCs), have shown promise in treating AKI due to their anti-inflammatory, anti-apoptotic, angiogenic, antioxidative stress, anti-fibrotic, and autophagy-regulatory properties [55]. Various animal studies and clinical trials have demonstrated the potential of MSCs in AKI treatment [55-56]. While autologous MSCs take longer to prepare but have low immunogenicity and no risk of infection, allogeneic MSCs can be produced rapidly in large quantities in vitro. New developments include acellular exosomes and MSCs derived from human pluripotent stem cells [57]. Although MSCs are relatively safe, more research is needed to address therapeutic safety levels and overcome barriers to clinical application.

Alkaline phosphatase (AP), an endogenous enzyme, is emerging as a targeted molecular therapy for S-AKI. Pickkers et al., in their double-blinded randomized control trial, found that AP is a promising new treatment for patients with severe sepsis or septic shock [58]. AP exerts detoxifying effects through the dephosphorylation of endotoxins, and administration of bovine AP has been shown to attenuate urinary excretions of tubular injury markers. In septic patients with AKI, treatment with AP improved overall renal function, as evidenced by enhanced endogenous creatinine clearance and reduced requirement and duration of dialysis [58].

Targeted cell therapies for S-AKI address the dysregulation in the host's immune response. While preclinical models in immunotherapeutics have shown encouraging results, clinical implementation remains challenging [56]. Advancements in genomics, proteomics, and metabolomics are helping to identify sepsis endotypes and treatable traits, potentially impacting the development of personalized treatment approaches. Artificial intelligence and machine learning are being utilized to analyze extensive clinical and immune data, guiding the discovery of new treatable traits [59]. The role of host metabolism and microbiome in sepsis is also being explored, emphasizing the importance of understanding patients' immunologic and metabolic status to identify risk factors and therapeutic targets [60].

Long-term outcomes and follow-up

The outcomes of S-AKI are of particular concern, as prevention is often not possible due to patients presenting with AKI already in progress. Our understanding of recovery patterns after AKI and their implications for long-term outcomes remains limited [61].

S-AKI is a severe condition strongly linked to poor clinical outcomes. Among critically ill patients with AKI, those with S-AKI face a higher risk of in-hospital death (odds ratio: 1.48) and significantly longer hospital stays compared to AKI from other causes (37 vs. 21 days) [62]. The requirement for in-hospital RRT is strongly associated with hospital mortality. However, patients who achieve renal recovery after S-AKI show dramatically improved survival rates.

Recovery from S-AKI can follow various patterns, including early sustained reversal, late sustained reversal, relapse with recovery, relapse without recovery, and never reversed. Factors influencing recovery may include preexisting renal functional reserve, severity and duration of AKI, and repetitive AKI episodes [63].

Compared to nonseptic AKI, S-AKI is associated with a higher acuity of illness. Patients with more severe AKI, as defined by RIFLE criteria, are more likely to have higher Acute Physiology and Chronic Health Evaluation II (APACHE II) scores [64]. Sequential organ failure assessment scores are also typically higher in patients with S-AKI compared to nonseptic AKI [64]. S-AKI patients often exhibit more abnormalities in markers of inflammation and blood biochemistry and are more likely to require mechanical ventilation, hemodynamic support with vasoactive therapy, and larger volumes of fluid for resuscitation [65].

Regarding renal function recovery, one study found complete recovery in 95.7% of 315 S-AKI patients, with a mean time for complete recovery of 10.1 ± 8 days [61]. The Beginning and Ending Supportive Therapy for the Kidney study showed similar rates of dependence on chronic RRT for septic AKI (5.7%) versus nonseptic AKI (7.8%) patients [64].

Mortality rates are significantly higher for patients with S-AKI compared to those with nonseptic AKI. ICU mortality rates were reported as 19.8% for S-AKI versus 13.4% for nonseptic AKI, while in-hospital mortality rates were 29.7% versus 21.6%, respectively [61]. Mortality rates increase stepwise with AKI severity, as defined by the RIFLE criteria. For instance, mortality was significantly higher in patients with S-AKI for AKI-AKIN stage 3 (64.1%) compared to AKI-AKIN stage 1 (34.6%) [64].

The timing of S-AKI onset is crucial. Observational data suggest that injury during S-AKI occurs early in the course of critical illness, often within 24 hours of ICU admission [54]. A large recent cohort study found that 68% of 5,443 patients with septic shock had evidence of AKI within 6 hours after presentation [65]. Patients showing evidence of kidney function recovery or improvement in their RIFLE category within 24 hours after presentation had better survival compared to those with no AKI or persistent AKI beyond 24 hours [66]. Factors associated with early recovery from AKI within 24 hours include younger age, early appropriate antimicrobial therapy, lower APACHE II scores, and community-acquired infection [62]. Conversely, the development of AKI later during an episode of sepsis has been associated with worse clinical outcomes and increased mortality rates (76.5% compared with 61.5% in early AKI) [66].

## Conclusions

S-AKI presents a complex interplay of pathophysiological mechanisms, including hemodynamic alterations, microcirculatory dysfunction, inflammatory responses, and oxidative stress. Early detection and timely intervention are crucial to improving outcomes, but the management of S-AKI remains challenging due to its multifaceted nature. Current treatment strategies emphasize supportive care, fluid management, and the use of renal replacement therapy, while emerging therapies, including pharmacological interventions and stem cell treatments, offer potential for improved outcomes. Ongoing research is essential to better understand S-AKI and develop more effective therapeutic approaches.
